# Real Time Blood Testing Using Quantitative Phase Imaging

**DOI:** 10.1371/journal.pone.0055676

**Published:** 2013-02-06

**Authors:** Hoa V. Pham, Basanta Bhaduri, Krishnarao Tangella, Catherine Best-Popescu, Gabriel Popescu

**Affiliations:** 1 Quantitative Light Imaging Laboratory, Department of Electrical and Computer Engineering, Beckman Institute for Advanced Science and Technology, University of Illinois at Urbana-Champaign, Urbana, Illinois, United States of America; 2 Department of Pathology, Christie Clinic and University of Illinois at Urbana-Champaign, Urbana, Illinois, United States of America; 3 College of Medicine, University of Illinois at Urbana-Champaign, Urbana, Illinois, United States of America; Tufts University, United States of America

## Abstract

We demonstrate a real-time blood testing system that can provide remote diagnosis with minimal human intervention in economically challenged areas. Our instrument combines novel advances in label-free optical imaging with parallel computing. Specifically, we use quantitative phase imaging for extracting red blood cell morphology with nanoscale sensitivity and NVIDIA’s CUDA programming language to perform real time cellular-level analysis. While the blood smear is translated through focus, our system is able to segment and analyze all the cells in the one megapixel field of view, at a rate of 40 frames/s. The variety of diagnostic parameters measured from each cell (e.g., surface area, sphericity, and minimum cylindrical diameter) are currently not available with current state of the art clinical instruments. In addition, we show that our instrument correctly recovers the red blood cell volume distribution, as evidenced by the excellent agreement with the cell counter results obtained on normal patients and those with microcytic and macrocytic anemia. The final data outputted by our instrument represent arrays of numbers associated with these morphological parameters and *not* images. Thus, the memory necessary to store these data is of the order of kilobytes, which allows for their remote transmission via, for example, the cellular network. We envision that such a system will dramatically increase access for blood testing and furthermore, may pave the way to *digital hematology.*

## Introduction

Blood is a specialized fluid that circulates through the heart and blood vessels, delivering necessary nutrients and oxygen to the cells and carrying away metabolic waste products [1]. Hematologic disorders and various diseases of other organs are reflected in specific findings from *blood tests*. Existing clinical technologies used to characterize blood samples such as impedance counters and flow cytometers are often accurate and offer high throughput. However, they are expensive and only provide limited information about the red blood cells: typically the mean corpuscular volume (MCV) and hemoglobin concentration (MCHC). Based on these parameters, the instrument flags abnormalities which require further investigation. Detailed morphological analysis is performed manually by a trained physician who visually assesses the *stained* blood smears through the light microscope. The process of staining is time and labor intensive, and requires a dedicated infrastructure, i.e., specialized instruments, dyes, as well as trained personnel [2]. It is precisely the absence of technology and clinical expertise that prevents blood testing from becoming universally available. Economically-challenged countries as well as rural areas in more developed countries have limited access to blood testing. Testing the blood from transfusions is a problem of global importance: 39 out of the 159 countries, which collect 92 million blood donations every year, were not able to run blood screens [3].

To address this problem, researchers have made great progress towards decreasing the cost of the imaging instruments by taking advantage of commercial technology, such as cell phone cameras. Thus, novel light microscopy designs have resulted in miniaturized and inexpensive devices for cell imaging in low resource settings [4], [5], [6], [7], [8], [9]. In particular, the combination of microscopy and microfluidics promises to commoditize imaging instruments and even convert them into disposable accessories [10]. However, the information provided by such instruments has been *qualitative*, i.e., the output data consist of images that require further analysis by trained staff. While in principle these data can be transmitted for remote diagnosis, a principle known as *telepathology* [11], the transfer of large image files is prohibited in economically challenged areas.

Here we propose a different technological approach for providing blood testing at the global scale. Instead of focusing primarily on lowering the cost of the instrument, we prioritized the quality of the data collected to attain computer-controlled, *quantitative* analysis. Thus, we developed a highly sensitive and *quantitative* instrument that operates in *real time* without human input. To our knowledge, this is the first real-time QPI system ever reported. The image rendered is the result of optical interference and provides *nanometer scale* information about the red blood cell profile, which translates into highly sensitive measurement of the volume and morphology. We used a highly parallelized image processing algorithm developed in house, which takes advantage of the computing power of graphic processing units (GPU), often employed in video games. This combination of novel optics and computation allows us to extract morphological parameters at the single cell level from the entire field of view (1 megapixel) in less than 25 ms. Thus, a thousand cells can be analyzed in less than 5 minutes. Importantly, the data outputted by our instrument represent arrays of numbers (text files), which are the result of thousands of images. Unlike the images they are distilled from, these data files require only kilobytes of memory per patient and can easily be transmitted wirelessly over the cellular network. This aspect, together with the fact that the blood necessary for this test can be obtained via a simple finger prick (akin to that in a glucose test), we envision that our instrument can operate in areas where clinical expertise and infrastructure are absent.

The paper is structured as follows. First, we describe the principle of our quantitative phase imaging (QPI) method, white light diffraction phase microscopy (wDPM) and the real time processing based on Compute Unified Device Architecture (CUDA). We demonstrate the performance of the system on blood testing patients suffering from *macrocytic* and *microcytic* anemia and perform a quantitative comparison between the MCV values provided by our method and the current clinical state of the art instruments. We illustrate the clinical capability of our instrument by presenting cell parameters that are currently not available from cell counters: RBC surface area, thickness, sphericity, minimum cylindrical diameter, equivalent diameter. Finally, we summarize and discuss the relevance of our results for universal blood testing.

## Materials and Methods

### Ethics Statement

The studies have been performed in the United States in accordance with the procedure approved by the Institutional Review Board at University of Illinois at Urbana-Champaign (IRB Protocol Number: 10571). All the blood samples used in our experiments were discarded clinical specimens, i.e., they were medical waste, as all the clinical studies needed for the patient care were completed by the clinical laboratory. All the blood specimens used in this research project were procured after securing a general consent form that was signed by the patients. The general consent form allows the discarded tissue to be used for educational and research purposes.

### Blood Sample Preparation

Blood sample from the hospital is first diluted in PBS solution with 0.1% albumin to a concentration of 0.2% whole blood in solution. A sample chamber is created by punching a hole in double sided scotch tape and sticking one side of the tape onto a cover slip. The sample is then pipetted into the chamber created by the hole and it is sealed on the top using another cover slip. The cells are allowed to settle for 5 minutes prior to measurement.

Since the index of refraction of hemoglobin may change from patient to patient [12], [13], [14], we accounted for the refractive index change according to the mean cell hemoglobin concentration that was measured independently by lysing the cells for each patient. Thus, the index of refractive depends linearly on the hemoglobin concentration of red blood cells as 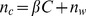, where β is the refractive increment, *C* the hemoglobin concentration of the cell, and *n_w_* is the refractive index of water. For different patients, the refractive indices of RBCs were corrected accordingly to their mean cell hemoglobin concentration (MCHC). For this study, we use the MCHC values provided by the impedance analyzer. This hemoglobin concentration can be also measured by other methods [15], [16], [17], [18].

### Quantitative Phase Imaging using White Light Diffraction Phase Microscopy (wDPM)

We developed an integrated hardware-software system that can measure in real-time detailed information from thousands of cells in a blood film. The underlying principle of the microscope is *quantitative phase imaging* [19], in which we retrieve the optical pathlength map associated with the blood film. Because the optical pathlength (or phase) contains information about both the sample refractive index and thickness, QPI has been used to provide measurements of red blood cell volumes [20], cell dry mass [21], [22], [23], [24], [25], dynamics [26], [27], [28], [29], [30], [31], cell tomography [32], [33], [34], [35], tissue scattering [36], [37], [38]. QPI has attracted increasing scientific interest in the past decade especially because it can study structure and dynamics *quantitatively*, with *nanoscale* sensitivity, and without the need for labeling with contrast agents. Various QPI methods have been proposed to satisfy particular requirements in terms of acquisition rate, transverse resolution, temporal and spatial pathlength sensitivity (for a review, see Chapter 8 in Ref. [19]). We chose white light diffraction phase microscopy (wDPM), which is a highly stable QPI method developed in our laboratory, which can operate at high acquisition rates [39].

### Morphological Parameters of Single Red Blood Cell

The first parameter calculated is the projected area (PA), which can be easily obtained by multiplying the number of pixels of each cell with the pixel area. PA then can be used to calculate the *equivalent circular diameter* with an assumption that the projected area of a RBC is a circular disk. In order to obtain other 2D and 3D morphological parameters, the phase map 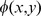 is converted to a height map 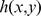 as 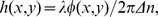 where 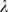 is the wavelength of the light source and 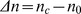 is the refractive index difference between RBCs and the surrounding medium. Once the height information is retrieved, the *volume* of each cell is calculated by integrating the height map over the projected area as 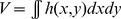. The *surface area* of individual cells is determined using Monge parameterization [40], where the contribution of each pixel element *dA* can be calculated as 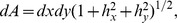 where *dx* and *dy* are the width and height of each pixel, and *h_x_* and *h_y_* are the gradients along the x and y directions, respectively. The surface area of each cell is the sum of all the area elements and the projected area, as the cell lays flat on the coverslip. From the surface area and volume, we calculate parameters such as sphericity (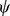) and minimum cylindrical diameter (*MCD*). The sphericity 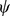 of RBCs was first determined as an important parameter by Canham and Burton [41]. It is defined as the ratio between the surface area (SA) of a sphere with the same volume as the cell, to the actual surface area of the cell. The sphericity index is calculated as 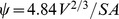 and has values ranging from 0 to 1. The MCD, introduced by Canham and Burton, is a theoretical parameter that predicts the smallest capillary diameter that a given RBC can squeeze through and, thus, is clinically significant. Furthermore, for each cell, we are able to calculate simultaneously many other independent parameters [15], including: perimeter, circular diameter, eccentricity, minimum, maximum, and mean thickness, circularity, integrated density of the cell, and kurtosis, skewness, and variance for cell height distribution. Given the vast amount of information available about each cell, this may open up opportunities to study and characterize abnormal cells and diseases that would otherwise be difficult or impossible to detect in an impedance counter or manually in a smear.

In our RBC measurements, we used a threshold on the calculated volume to exclude non-RBC cells. We set the minimum volume for RBC to 20 fL to exclude platelets from the analysis. Reticulocytes account for about 1 percent of red blood cells, contain ribosomal RNA, and are slightly larger than mature RBCs. It has been shown that phase shift information discriminates immature red blood cells from mature [42]. It is possible to add this feature to our system, but for the results presented in this manuscript, we did not separate reticulocytes from mature RBCs.

## Results

### Light Microscopy System Performs Blood Tests with Single Cell Parameters

**Figure 1:**
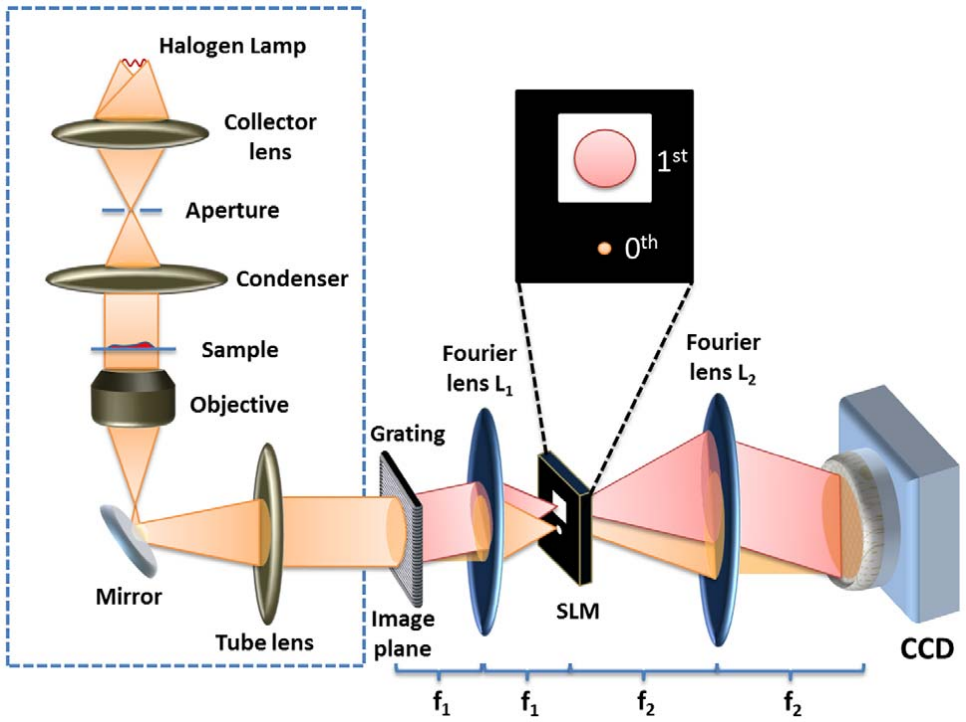
The wDPM setup. A grating is placed at the image plane of a commercial microscope (the dashed box) to create different diffraction orders. Filter masks are projected onto a spatial light modulator (SLM) placed at the back focal plane of a Fourier lens L1 to low pass filter the 0th order beam (reference) and allow the entire 1st order beam (imaging field) to pass through. Another Fourier lens L2 recombines the two beams to create an interferogram on a CCD. A Hilbert transform is then used to reconstruct the phase information from the interferogram.

The wDPM layout is shown in Fig. 1 and described in more detail in Text S1. Briefly, the image field outputted by an existing inverted microscope (inside the dash box in Fig. 1) is projected onto a grating, which splits the beam into two components, the 0^th^ and 1^st^ order (all other diffraction orders are filtered out). These two light paths form a very compact interferometer that generates fringes at the CCD plane. In order to transform the 0^th^ order of diffraction into the reference field of the interferometer, we spatially filter it at the back focal plane of the Fourier lens L_1_. This is achieved by using a liquid crystal spatial light modulator (SLM) that has such a particular transmission function to low-pass filter the 0^th^ order and transmit the 1^st^ order entirely (see inset in Fig. 1). Lens L_2_ reconstructs the image at the CCD plane, which now records interference fringes and, thus, optical pathlength information from the specimen.

Unlike off-axis digital holography methods, where there is a compromise in space-bandwidth product, our system performs the measurement at the image plane. Thus we can preserve optical resolution without sacrificing a large number of pixels. In our case, the minimum number of pixels required per fringe is 3. Thus, the space-bandwidth sacrifice is not significant, especially today, when 10 megapixel CMOS sensors are easily available and inexpensive.

### Real-time Analysis using CUDA

Typically, in order to obtain the pathlength map from an acquired *interferogram*, QPI involves off-line post processing. *Off-axis* methods, including wDPM (see Chapter 8 in Ref. [19] for a classification of QPI techniques), require processing in two steps: i) a spatial Hilbert transform to retrieve the phase from the interferogram and ii) an *unwrapping* algorithm to reconstruct the true phase information from the measured *wrapped* values, which are between –π to +π. These are time-consuming operations. For example, a serial C-code implementation of the whole process requires approximately half a second to process for a one-megapixel image on a current personal computer. Such processing times are unsuitable for the high-throughput required in blood testing. However, Compute Unified Device Architecture (CUDA) model based algorithms for phase unwrapping can improve on the traditional, serial method by a factor of 50 or more [43].

**Figure 2:**
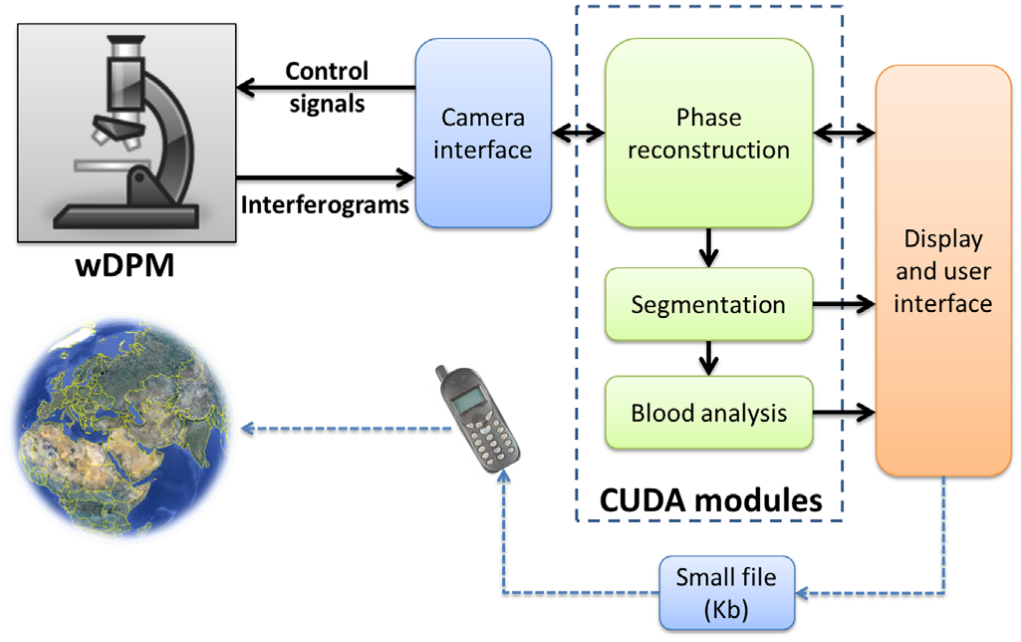
Diagram of the system. The imaging system wDPM is communicated to the user through the camera interface. User can control several parameters like exposure time, image size, etc. Several CUDA based modules are implemented to speed up the processing: The phase reconstruction module recovers the phase information induced by the objects in the field of view; the segmentation module isolates and labels individual objects in the field of view; and the blood analysis module was designed specifically for blood smear analysis application to calculate different morphological parameters of red blood cells. All the results are computed and displayed in real time at the speed of up to 40 frames/s. The computed parameters can be saved in a small file and sent anywhere for remote diagnosis.

In order to achieve real time performance, we developed our parallel programming software based on NVIDIA’s CUDA model. Figure 2 shows the overall structure of our imaging instrument.

Recently, the computation power of GPU’s has increased significantly. In the NVIDIA’s CUDA programming environment, GPUs can be regarded as computation devices operating as coprocessors to the central processing unit (CPU) [44]. The main idea is to process computationally-intensive parts in parallel by using multiple computation units. The CUDA architecture consists of hundreds of processor cores that operate together to process different segments of the data set in the application. A CUDA program consists of both host code and device code. The host code is straight ANSI C code, which is used when there is little or no data parallelism, and the device code is used when there is a rich amount of data parallelism. Based on single-instruction, multiple-thread (SIMT) architecture [44], CUDA maps a single kernel to a grid of threads to process different input data simultaneously. Threads are organized into blocks of up to three dimensions and the blocks are then organized into a one-dimensional or two-dimensional grid of thread. All threads in a block can synchronize their execution but two threads from different blocks cannot cooperate. All threads will execute the same instruction but on different input data identified by their thread indices and block indices.

**Figure 3:**
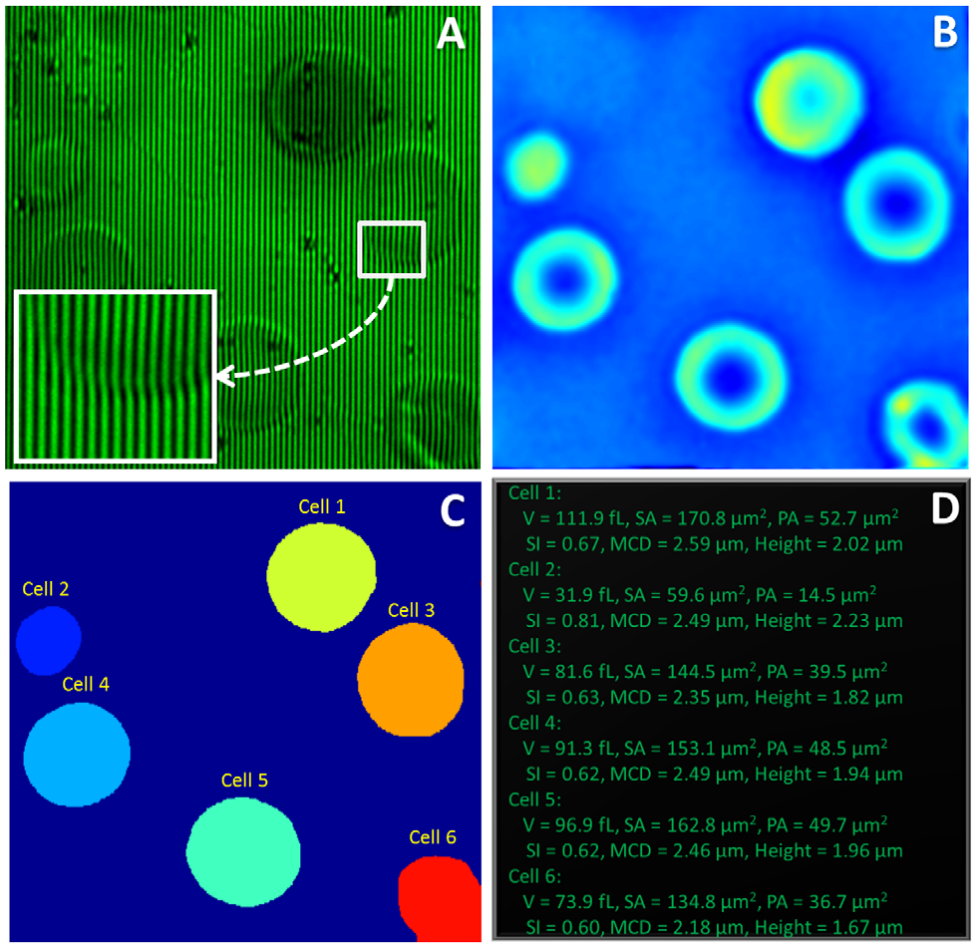
Phase image reconstruction and processing procedure. (**A**) An interferogram acquired from the wDPM system; (**B**) Reconstructed phase map; (**C**) The output of the segmentation module; (**D**) A snapshot of the screen visualizing different parameters of each red blood cell including volume (V), surface area (SA), projected area (PA), sphericity index (SI), minimum cylindrical diameter (MCD) and the mean height.

Figure 3 presents images illustrating different steps of our software. We tested the program on a Windows machine with Intel® Core™ i5 CPU with clock rate of 3.2 GHz and 8 GB RAM memory. We use NVIDIA® GeForce® GTX 570M GPU which supports CUDA programming. First, interferograms (Fig. 3A) from the wDPM imaging system are captured using Hamamatsu Orca Flash camera. Our program controls the camera (initialize, set parameters like image size, exposure time etc.) and acquires the interferogram image using the camera software development kit (SDK) provided by Hamamatsu Photonics. The inset in Fig. 3A zooms in a portion of the interferogram to show the high fringe contrast of our interferogram and illustrate the bending of the fringes due to the presence of a red blood cell. The interferogram is then transferred to the phase reconstruction module (Fig. 2) to be processed to get the phase image (Fig. 3B). More details on the algorithm and implementation of this module can be found in [43]. The phase image is then displayed on the screen using openCV library [45] for visualization. At the beginning of each measurement, we perform a background subtraction, which removes the effects of inherent dust and aberrations along the optical path. The efficacy of the phase background subtraction is evidenced by comparing Fig. 3A, where many dirt particles are present, and Fig. 3B obtained after this correction.

Next, the segmentation module first applies a simple global threshold to the phase image to separate the objects from the background. This will give a map where 0′s pixels of value zero are background pixels and other pixels are object pixels which are labeled by numbers corresponding to the pixel indices in the pixel array. In the next step, since there are several objects, e.g. red blood cells, in the field of view, in order to have quantitative information about each object, we need to segment and label each object individually. This process is called connected component labeling problem. It is an important problem appearing in many fields of research like computer vision for segmentation; cellular automata (CA) models used for different kinds of simulation in physics; mathematics and biology. Since the early 1970s, numerous approaches for connected component labeling have been introduced [46], [47], [48]. Most of these approaches are suitable for sequential processing. Recently, with the introduction of GPUs with interfaces as CUDA [44] or OpenCL [49], some parallel algorithms of graph component labeling with GPUs have been developed [50], [51]. In this paper, we adapted the “label equivalent” algorithm in [51] to develop our image processing tool. Fig. 3C shows the label map identifying different red blood cells in the phase image. Finally, different parameters are calculated for each cell and displayed as shown in Fig. 3D. Details of our implementation on connected component labeling and calculation of RBCs’ morphology parameters are discussed further in the Text S1 and Figures S1 and S2.

With our current GPU graphic card (GTX570), we can acquire images and perform all the processing of up to 40 frames/second, with the images of size 1024×1024 pixels and this rate can be increased when more powerful GPU cards are used. Movie S1 shows a video that illustrates the real time blood testing.

### Clinical Studies of Macrocytic and Microcytic Anemia Patients

We performed a clinical study on blood samples from patients with normal blood, macrocytic, and microcytic anemia. The blood samples were provided by Provena Covenant Medical Central laboratory. All specimens were handled according to safety regulations by the Institutional Review Board at the University of Illinois and Provena Covenant Medical Center. We compared our measurement results with those from the clinical Coulter impedance counter. We measured 6,181 RBCs of the normal patient, 8,442 RBCs of the patient with microcytosis anemia disease, and 4,535 RBCs of the patient with macrocytosis disease.

**Figure 4:**
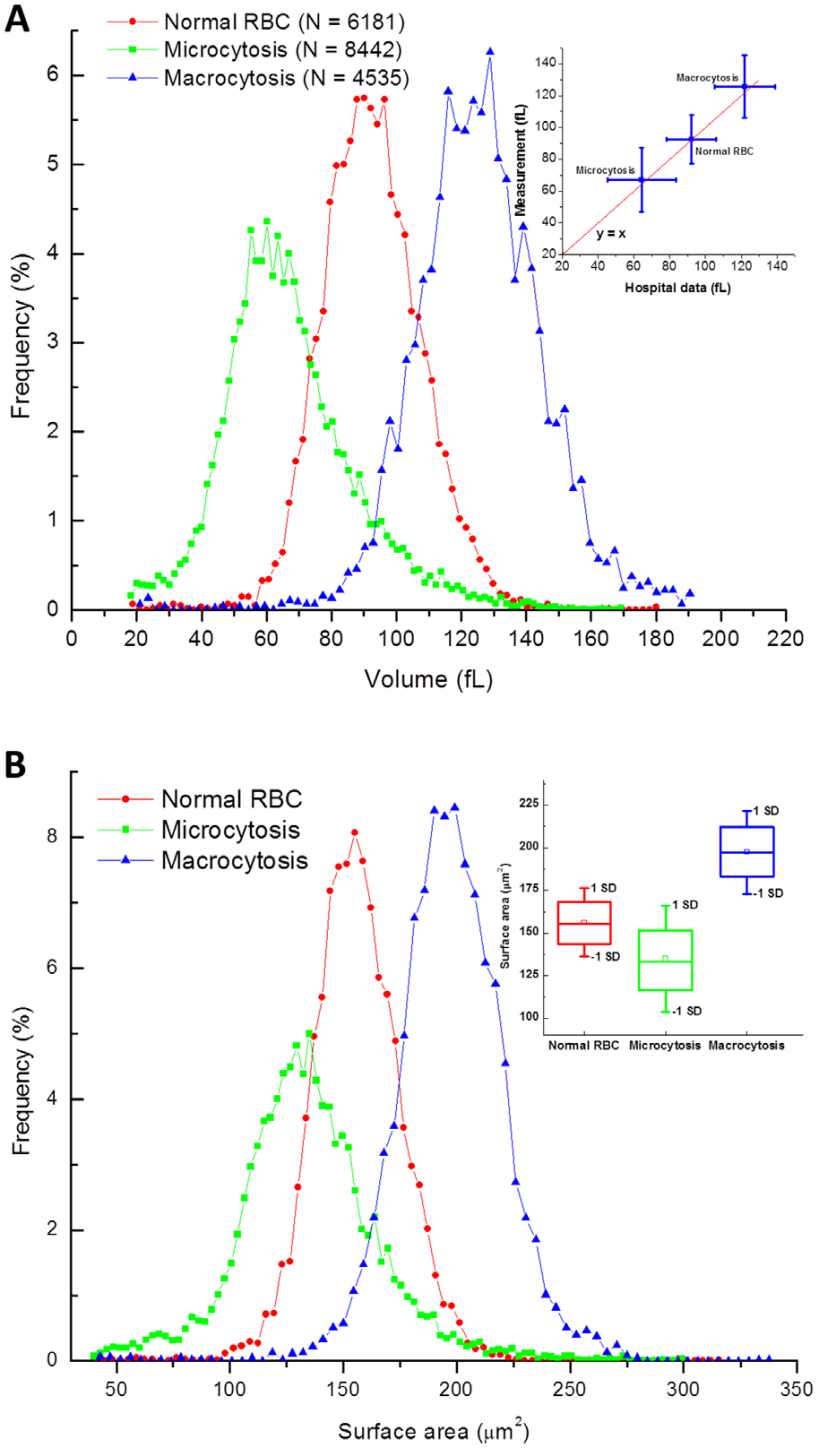
Morphological parameter distributions of red blood cells of different patients. (**A**). Red blood cell volume distribution of a healthy patient (N = 6,181 cells) and patients with microcytic (N = 8,442 cells) and macrocytic (N = 4,535 cells) anemias. The inset compares the measured results with the results obtained from a Beckman Coulter counter on the same samples with vertical and horizontal error bars are standard deviations from the measurement data and the Beckman counter, respectively. The red line illustrates the line y = x, of perfect agreement; (**B**). Red blood cell surface area distribution of the three patients. The inset elaborates the statistical information of the distributions with the range within one standard deviation, the boxes indicating the interquartile range (IQR) and the square symbol showing the median.

Figure 4A shows the distributions of RBCs’ volumes for three patients. The MCV values for normal, microcytic and macrocytic patient are 92.5 fL, 67.4 fL and 125.6 fL, respectively. These numbers agree very well with the data acquired by the clinical impedance counter, which are 92.2 fL, 64.4 fL and 121.8 fL, respectively. The inset in Fig. 4 illustrates the comparison between our measured data and the data from the clinical impedance counter. Another important RBC index is the red blood distribution width (RDW), which is the width of the distribution curve of RBCs’ volumes and equals the ratio between the standard deviation and MCV of the blood sample. Higher RDW values indicate greater variation in size. RDW can be useful in early classification of anemias because it becomes abnormal earlier in nutritional deficiency anemias than any of other red cell parameters, especially in case of iron deficiency [52], [53], [54]. RDW is also useful in identifying red cell fragmentation, agglutination, or dimorphic cell populations [54]. If anemia is observed, RDW test results are often used together with MCV results to determine the possible causes of the anemia. An elevated RDW (red blood cells of unequal sizes) is known as *anisocytosis*. We can see that the measured data matches very well with the mean (three center points) and standard deviation (error bars in the inset). Specifically, the red blood cell distribution width (RDW) for normal, microcytic and macrocytic patients are 16.5%, 30.1% and 15.6%, respectively, which agree with the data from the clinical analyzers (15%, 29.7% and 13.9%, respectively). In addition, the anisocytosis 3+ disease (high RDW) of the microcytic patient was confirmed with our measurement. Figure 4B shows, as expected, that the cell surface area increases from microcytic, to normal, to macrocytic cells.

**Figure 5:**
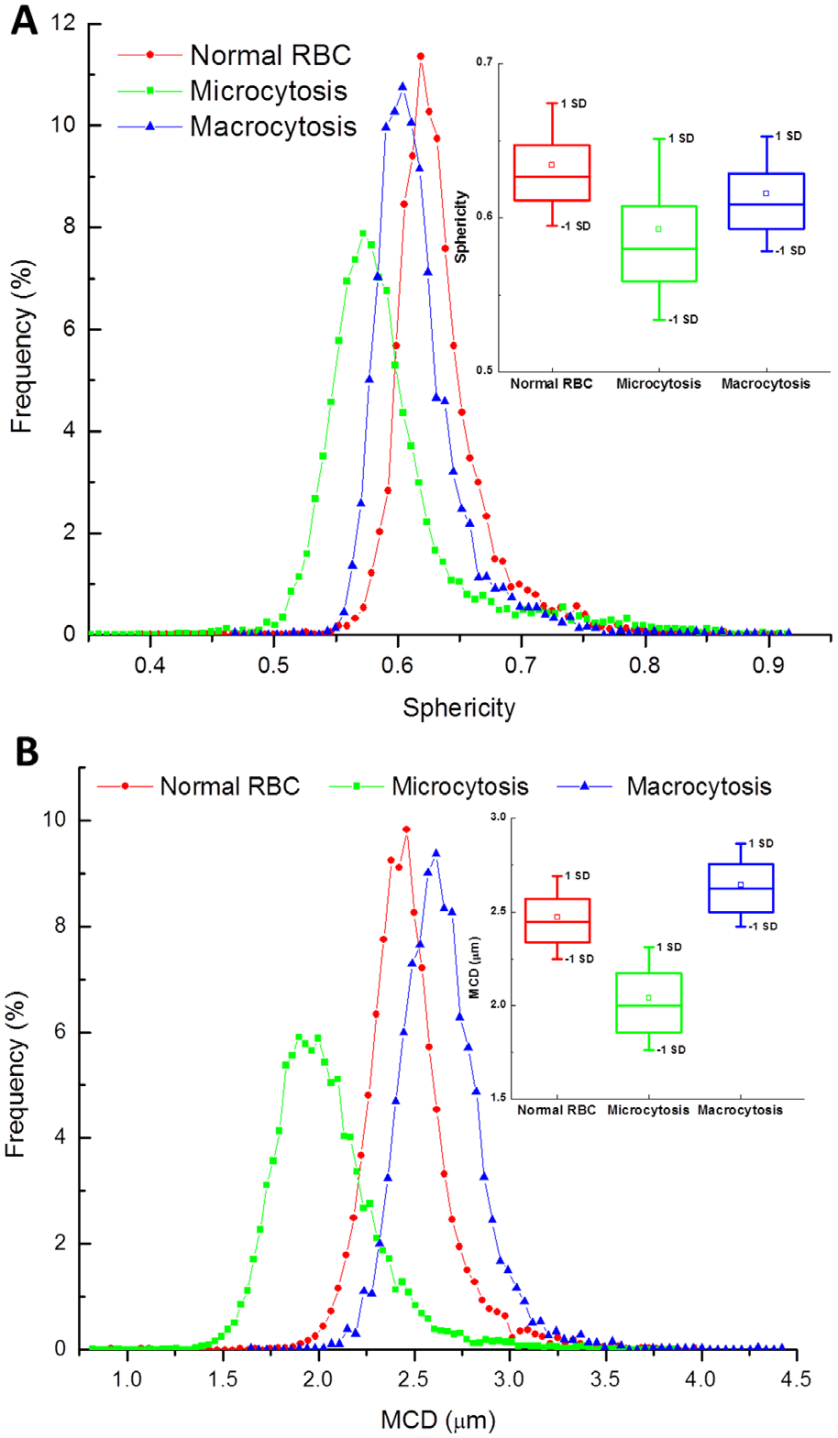
Examples of other morphological parameters of red blood cells. **A**. Sphericity index distribution; **B**. Minimum cylindrical diameter distribution.

The relationship between the surface area and volume determines the morphology of the cells. Our system is able to characterize this morphology in great detail. Figure 5 shows the results in terms of the sphericity index and mean cylindrical diameter. Interestingly, our measurements indicate that the normal population is characterized by the highest average sphericity. On the other hand, the MCD shows the expected trend of monotonous increase from microcytic to macrocytic cells. Current automated counters cannot provide these parameters. Thus, a pathologist has to manually examine a smear to confirm diagnosis of *spherocytosis*. Our system provides this diagnosis quantitatively using the sphericity index for single cell level as well as the population statistics. The insets in the two figures illustrate statistical information of the distributions of corresponding cell parameters of each patient with the range indicate standard deviation, the boxes show interquartile range (IQR) with the means at the middle lines and the square symbol indicate the median of the corresponding parameters. Finally, we present the distributions of red blood cell equivalent diameter and average cell height for the three patients in Figs. S3 and S4, respectively.

## Summary and Discussion

We demonstrate a quantitative phase imaging system dedicated to blood screening, which reconstructs phase images, analyzes and calculates a number of morphological parameters of red blood cells at single cell level, all in real time. The system is capable of very high throughput imaging and allows analyzing easily thousands of cells per sample. In terms of anemia, our technique can also count and compare cell numbers in a given volume of sample if necessary We measured blood samples of a healthy patient and patients with macrocytic and microcytic anemia. The resulted MCV distributions show an excellent agreement with the results from Beckman-Coulter counter. Furthermore, we show the results of several other morphological parameters, which are unavailable with the automatic analyzers used currently in clinical settings.

Our system provides a powerful and robust blood screening utility that can aid pathologists interested in performing remote diagnosis or screening. The vast amount of information on the different diagnostic parameters, as well as the high throughput and real time imaging provide a viable solution for removing economically-driven discrepancies in blood testing and screening. Currently, our throughput is still below that of flow cytometers, mainly due to manual control of microscopy stage movement. However, this acquisition rate is sufficient to provide practical clinical output in reasonable time, while providing much richer information on the cell morphology, which in turn provides additional diagnostic information about blood disorders. This can potentially remove the laborious and time intensive manual evaluation of blood smears under the microscope. The throughput can be improved significantly by integrating automatic stage control in the software. Furthermore, our method can be combined with microfluidic channels to measure blood cells flow through the channel and, with the current speed of 40 frames per second, it is feasible to achieve comparable throughput with that of flow cytometers in the near future.

The results presented here are the proof of principle of our method, which combines quantitative phase imaging and real-time processing. Investigating different diseases presenting altered red blood cell number and morphology require clinical validation through analysis of a large number of patients. This work of characterizing red blood disorders is in progress in our laboratory. Studies of different specific diseases deal with identifying the set of parameters from our measurement that is best suited to maximize the sensitivity and specificity of diagnosis. Our data can be easily formatted according to standards such as FCS 3.1, and used with automatic flow cytometry. This approach will take advantage of the existing analysis software.

In terms of cost, the quantitative phase imaging module is relatively inexpensive compared with the cost of the microscope itself; thus upgrading existing microscopes will be the most cost-effective approach. The cost per test is very low, due to the lack of sample preparation which includes an inherent cost in staining materials and trained laboratory personnel time.

Operating in remote and under-developed areas also involves operation under tough conditions, such as temperature and humidity variations. Of course, working under these circumstances requires further testing. However, the intrinsic stability of our common path system promises to fulfill these constraints.

## Supporting Information

Figure S1 **Flowchart of the segmentation module.**
(TIF)

Figure S2 **An example of label equivalence algorithm: (A) Initial label; (B) Label map after the first Scanning function call; (C) After the first Analysis function call; (D) Final label map.**
(TIF)

Figure S3 **Red blood cell equivalent circular diameter distribution.**
(TIF)

Figure S4 **Red blood cell average height distribution.**
(TIF)

Text S1 (DOC)

Movie S1 **Recording of real-time analysis of a blood smear.**
(WMV)
