# Supplementary figures and images for: Real Time Blood Testing Using Quantitative Phase Imaging
Source: PLoS One. 2013 Feb 6;8(2):e55676. doi: 10.1371/journal.pone.0055676 (PMC3565969; doi:10.1371/journal.pone.0055676)

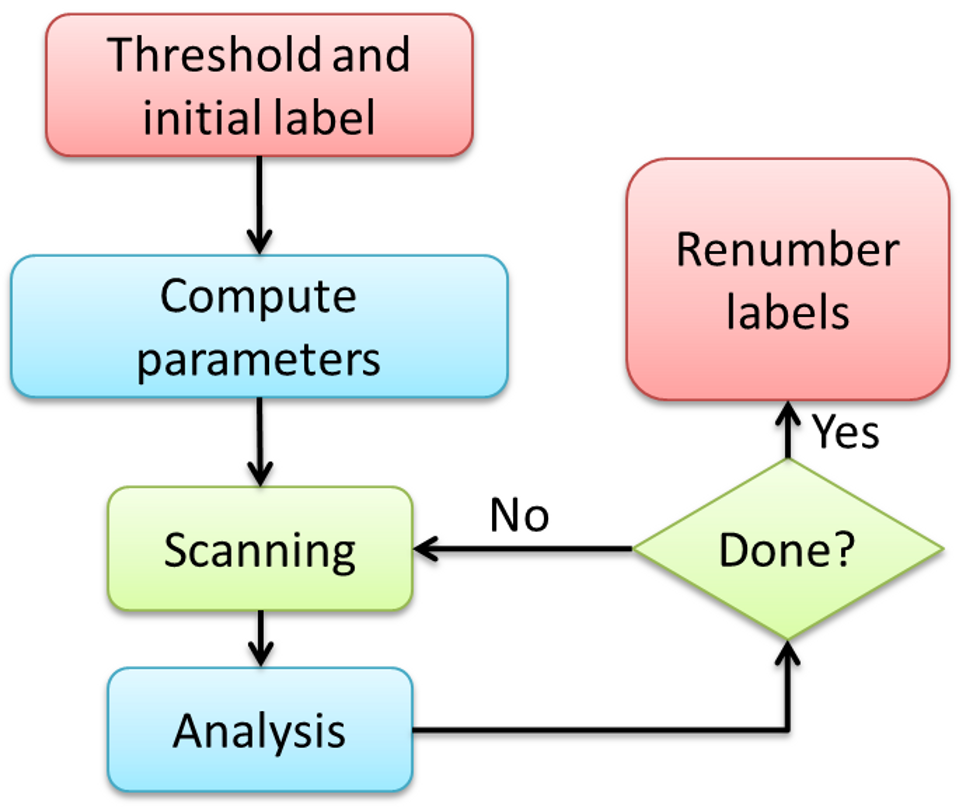

Supplement: Figure S1 — Flowchart of the segmentation module. (TIF) [file pone.0055676.s001.tif]

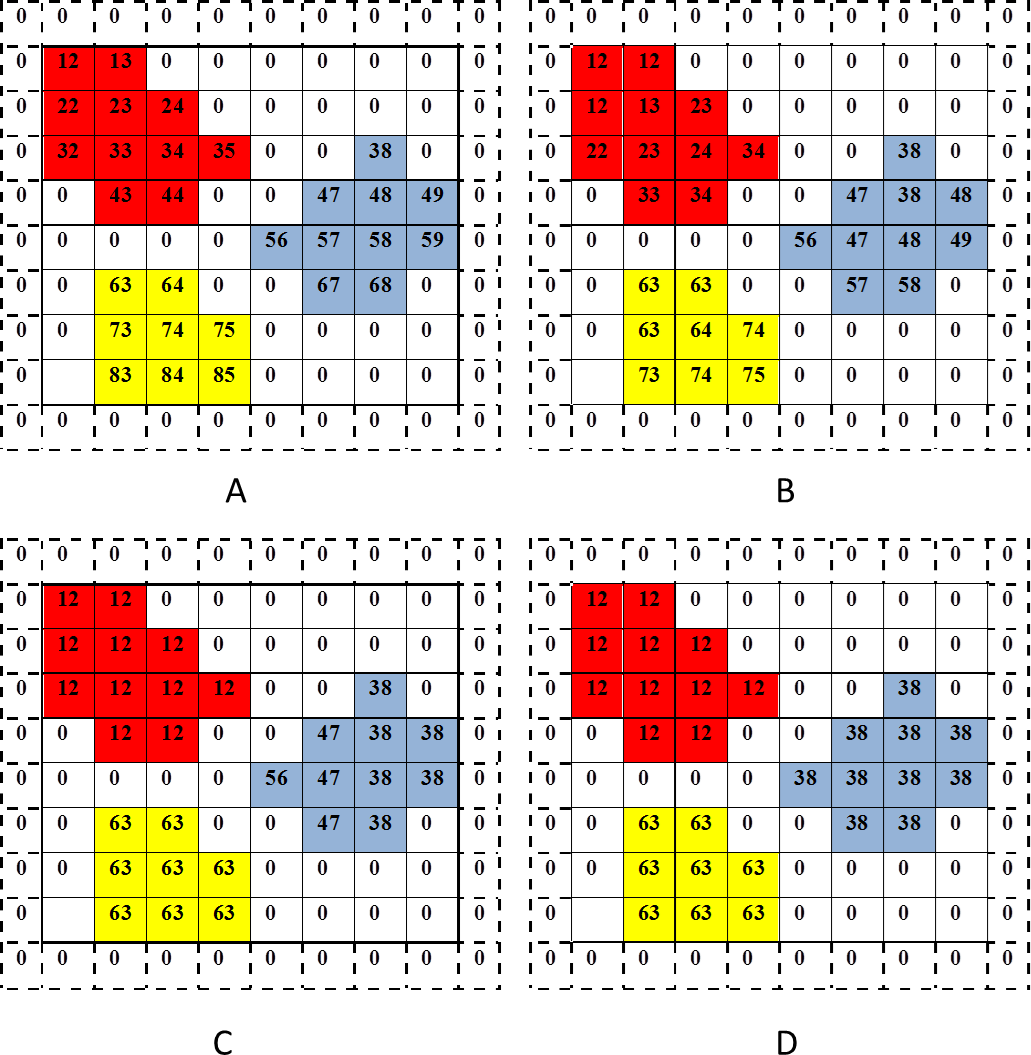

Supplement: Figure S2 — An example of label equivalence algorithm: (A) Initial label; (B) Label map after the first Scanning function call; (C) After the first Analysis function call; (D) Final label map. (TIF) [file pone.0055676.s002.tif]

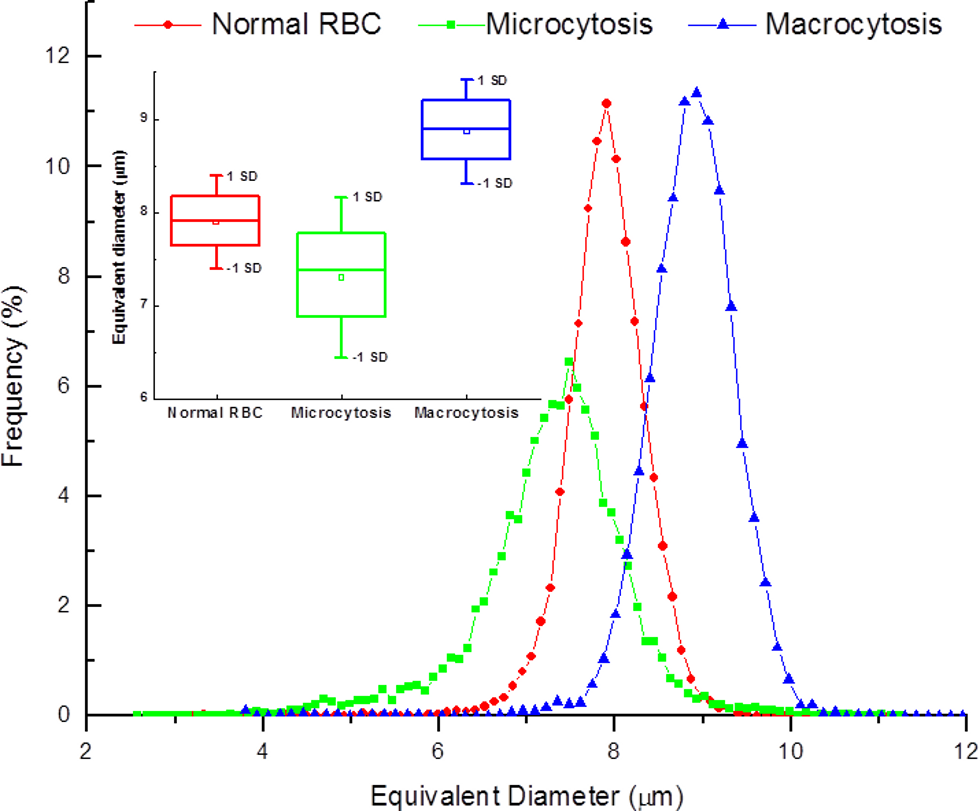

Supplement: Figure S3 — Red blood cell equivalent circular diameter distribution. (TIF) [file pone.0055676.s003.tif]

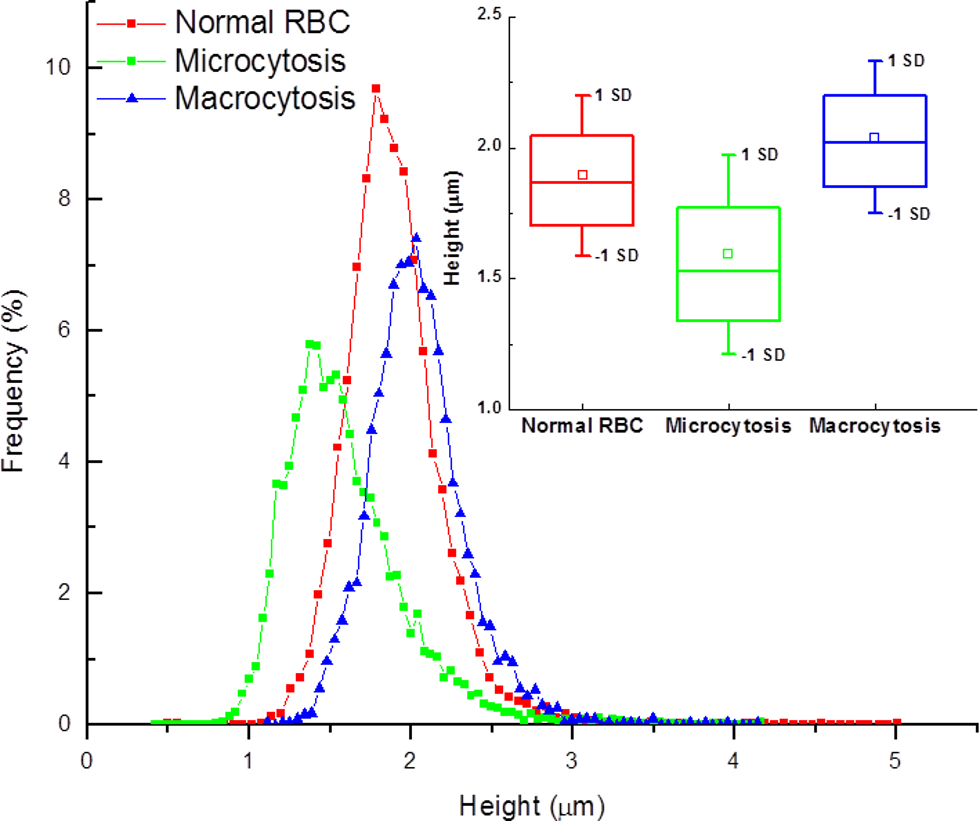

Supplement: Figure S4 — Red blood cell average height distribution. (TIF) [file pone.0055676.s004.tif]
