# Supplementary material for: Real Time Blood Testing Using Quantitative Phase Imaging
Source: PLoS One. 2013 Feb 6;8(2):e55676. doi: 10.1371/journal.pone.0055676 (PMC3565969; doi:10.1371/journal.pone.0055676)
Supplement: Text S1 — (DOC) [file pone.0055676.s005.doc]

Real time blood testing using quantitative phase imaging

**Hoa V. Pham,^1,*^ Basanta Bhaduri^1^, Krishnarao Tangella^2^, Catherine Best-Popescu and Gabriel Popescu^1^**

^1^ Quantitative Light Imaging Laboratory, Department of Electrical and Computer Engineering, Beckman Institute for Advanced Science & Technology University of Illinois at Urbana-Champaign, Urbana, Illinois, United States of America

^2^ Department of Pathology, Christie Clinic and University of Illinois at Urbana-Champaign

Urbana, Illinois, United States of America.

^3^College of Medicine, University of Illinois at Urbana-Champaign, Urbana, Illinois, United States of America

[*hoapham2@illinois.edu](mailto:*hoapham2@illinois.edu)

**Supplementary Information**

1. **White light diffraction phase microscopy (wDPM)**

Figure 1 shows the optical setup of wDPM which is implemented as an add-on module to a commercial inverted microscope (Axio Observer Z1, Zeiss). We used spatially coherent white light illumination, obtained from a halogen lamp commonly used in commercial microscopes. We closed down the condenser aperture such that the field is spatially coherent over the entire field of view. A diffraction grating located at the image plane of a microscope is used to generate diffraction orders, each containing full spatial and phase information of the sample. A mask is projected onto a SLM placed at the Fourier plane to filter the diffraction orders. The SLM was obtained from an Epson Powerlite S5 projector, having a pixel size of 13×13μm^2^ and contrast ratio of 400:1. The zero-order beam is spatially low-pass filtered using a circular mask so that it can be used as a reference beam. The whole +1 diffraction order is passed through and is used as the sample beam, and all the other orders are blocked. A second lens is then introduced to project the interferogram onto the camera (ORCA-Flash 2.8, Hamamatsu Photonics) plane for recording the interferograms. The grating period was chosen to be smaller than the diffraction-limited spot of the microscopic imaging system at the grating plane. In our setup, we used a transmission grating with groove density of 110 grooves per mm (Edmund Optics). All the lenses were achromatic to minimize chromatic dispersion. Throughout our experiments, the microscope was equipped with a bright-field 20X (0.8 NA) objective. The L1–L2 lens system gives an additional magnification of f2 / f1=2.5, such that the sinusoidal modulation of the image is sampled by 6 camera pixels per period. wDPM combines the benefits of high temporal sensitivity of common path and off-axis technique and high spatial sensitivity of white light illumination.

1. **Parallel label equivalence algorithm incorporating calculation of parameters**

The label equivalence algorithm is used to label different connected regions in an image (as a 2D array of pixels) with different numbers to uniquely identify different objects in the image. Figure S1 shows a diagram of the label equivalence algorithm and Figs. S2A-D illustrate an example to clarify steps in the algorithm.

First, the label map is padded with 0’s at the boundary to avoid boundary checking in the program code, which lead to divergence in the code and slow down the speed performance. Then, the function “*Threshold”* takes a reconstructed phase map as an input and applies a threshold to separate interested objects from the background. Each non-background pixel is assigned a label equal to its index in the image and this index is regarded as its initial reference. Fig. S2A shows the initial label map with zero padding. The volume, surface area contributions and pixel count (to be used to calculate projected area, average height and other parameters later) are then calculated and stored for each non-background pixel.

After the initial labeling, the main loop containing two main functions: “*Scanning*” and “*Analysis*”, are called iteratively in a loop until each object in an image is labeled to a unique identifier. These are done in parallel at each and every pixel. The first function chooses the smallest nonzero label value within five pixels: one central (the current pixel) and its four neighbor pixels. The chosen value will then be assigned to the current pixel. Figure S2B presents the label map after the first calling of the *Scanning* function. The second function performs relabeling the pixels. In this function, another inner loop is started at the current pixel to check if its current label (considered as its reference) matches with the index of that element. If not, the next iteration checks the label of the reference and so on until the match is found. Figure S2C illustrates the label map after the first call of the *Analysis* function. A binary global indicator is set to one if the label of any pixel in the image is changed in an iteration. This indicator is used to stop the loop when no further iterations are needed. Figure S2D shows the final label map where every pixel in a connected region is labeled the same number. Another job of the *Analysis* function is to compute different parameters of RBCs. At each pixel, the volume, surface area are calculated and during each iteration of the *Analysis* function, these parameters and pixel count of each cell will be accumulated in such a way that after the final iteration, the first pixel in each cell is associated with the total volume, surface area and the pixel count of the whole cell.

After the main loop finishes, we have a map with background pixels of value 0 and groups of pixels with different values for different groups, all pixels in the same connected region should have the same label values. Finally, we need to renumber the group starting from 1. Notice that the pixel with smallest index in a group has its label equal its index in the array. These pixels in different group will be used to identify and count the objects in the field of view. In order to avoid conflict since many pixels are processed at the same time, we use “*atomicAdd*” function in CUDA library to count the number of objects (connected components) in the field of view. This function increases a counter by one every time an objected is found and all the requests to execute the function are queued to avoid confliction. Figure 3C shows the label map as a result of the segmentation module with the input phase map in Fig. 3B. Here, each RBC is identified by a unique label number starting from one.

1. **Red blood cell volume calibration**

In this study, we compare our measurement results with those obtained from a Beckman Coulter counter used in the hospital. In order to compare our measurements with the clinical results, we first measured 2,000 RBCs of the normal patient and then normalize our mean cell volume to the MCV result achieved by the impedance counter. This calibration is necessary because cell counters do not measure cell volumes directly, but rather the volume of the *sphered* red blood cells*.*

1. **Other red blood cell parameters**

Figures S3 and S4 show two other morphological parameters of red blood cells: the equivalent circular diameter and the average height. The circular diameters were calculated from the projected area of the cells and the heights were averaged throughout the cell for each individual cell.
